# The Ferroptosis Molecular Subtype Reveals Characteristics of the Tumor Microenvironment, Immunotherapeutic Response, and Prognosis in Gastric Cancer

**DOI:** 10.3390/ijms23179767

**Published:** 2022-08-29

**Authors:** Xiao Xu, Na Zhou, Hongwei Lan, Fangfang Yang, Bowen Dong, Xiaochun Zhang

**Affiliations:** 1 Precision Medicine Center of Oncology, The Affiliated Hospital of Qingdao University, Qingdao 266003, China; 2 Qingdao Medical College, Qingdao University, Qingdao 266071, China

**Keywords:** ferroptosis, tumor microenvironment, stromal activation, tumor mutation burden, immunotherapy

## Abstract

Ferroptosis is a relatively new form of programmed cell death, which can enhance the efficacy of tumor immunotherapy by regulating the tumor microenvironment (TME). In the face of the dilemma of a great difference in the efficacy of immunotherapy for gastric cancer (GC) patients, the exploration of ferroptosis may assist us in predicting immunotherapy efficacy prior to treatment. The potential role of ferroptosis in TME still needs further elucidation. Based on ferroptosis-related genes (FRGs), we systematically evaluated ferroptosis molecular subtypes in gastric cancer. Additionally, the association between these molecular subtypes and the characteristics of TME was examined. A ferroptosis score was constructed to further explore the predictive efficacy of ferroptosis on the immunotherapy response in gastric cancer. There were also 32 other cancers that were evaluated. Three molecular subtypes of ferroptosis in gastric cancer were identified. The three immunophenotypes of tumor immune inflamed, immune excluded, as well as immune desert were mostly in agreement with the TME features of these three subtypes. The individual tumor genetic variation, TME characteristics, immunotherapy response, and prognosis could be assessed by a ferroptosis score. High ferroptosis scores in gastric cancer suggest stromal activation and immunosuppression. It is noted that tumors with a low ferroptosis score are characterized by extensive tumor mutations as well as an immune activation, which are associated with an enhanced immunotherapy response and an improved prognosis. This study reveals that ferroptosis plays an integral role in the regulation of the tumor immune microenvironment. The ferroptosis score may serve as an independent prognostic factor for GC and will deepen our understanding of the TME infiltration mechanisms as well as lead to more rational immunotherapy regimens.

## 1. Introduction

Gastric cancer is one of the five most common cancers worldwide [1,2], and although the incidence and mortality rates of gastric cancer have declined to varying degrees in recent years, the level of public health threat posed by gastric cancer is undeniably high. The development and progression of gastric cancer is complex and also involves multiple molecular mechanisms and complex signal transduction systems [2]. Differences in tumor aggressiveness, histopathological features, and the response to therapy exist among patients [3]. There has been a considerable increase in the survival benefits among advanced gastric cancer patients since immunotherapy has broken the previously monopolistic role of chemotherapy and targeted therapy [4,5,6,7]; however, the major challenge of immune checkpoint blockade therapy is the disparity in patient outcomes caused by the intricacy of the tumor microenvironment and the under-activation of the parasitifer immune system [8,9]. Consequently, there is an urgent need to develop more precise markers to provide insight into carcinogenesis and predicting the therapeutic response.

Ferroptosis involves a complex biological process caused by the disturbance of iron metabolism and the accumulation of reactive oxygen species (ROS) leading to lipid peroxidation [10,11]. Recent studies have reported that ferroptosis is vital for eliminating tumor cells and suppressing tumor development [12,13,14]. As a recognition signal, ferroptosis-associated lipid peroxides stimulate dendritic cells to recognize, phagocytose, and process tumor antigens before presenting them to CD8+ T lymphocytes. The CD8+ T cells inhibit tumor cell cystine uptake by releasing IFN-γ, which can activate cytotoxic T lymphocytes to enhance tumor immunotherapy [15,16,17]; thus, using ferroptosis to improve tumor immunotherapy by focusing on the tumor microenvironment may be a new and effective way to improve tumor immunotherapy.

Cancer relies on a complex tumor microenvironment to sustain growth, invasion, and metastasis [18,19]. Heterogeneous non-malignant cells, including cancer-associated fibroblasts (CAFs), tumor-associated immune cells (TICs), and vascular cells, form the tumor microenvironment [18]. The inflammatory state of the TME has now been shown to be essential for the initiation, progression, and metastasis of almost all solid tumors [8]. The epithelial-mesenchymal transition (EMT) in the TME was previously thought to be associated only with the invasive metastasis of cancer cells, but recent studies have identified EMT as an important mechanism of tumor drug resistance. Several studies have shown that growth factors, tumor microenvironment (e.g., hypoxia), and multiple oncogenic-related signaling pathways (e.g., the TGF-β signaling pathway, Notch signaling pathway, MAPK signaling pathway, and KRAS signaling pathway) can all initiate the EMT process [20,21,22]. Mariathasan et al. collected previous studies to construct a set of EMT marker genes: EMT1 (breast cancer) [23], EMT2 (urothelial carcinoma) [24], EMT3 (metastatic melanoma) [25], angiogenic signature [26], and WNT targets [27]. To specifically measure the TGF-β pathway activity in fibroblasts, they developed a pan-fibroblast TGF- response signature (Pan-F-TBRS) [28]. They collected a large number of patients with uroepithelial carcinoma from patients treated with an anti-PD-L1 drug (atezolizumab) and found that a good immunotherapy response was associated with a CD8+ T-effector cell phenotype and tumor mutational load (TMB). In fibroblasts, a signature of transforming growth factor (TGF-β) signaling was associated with a lack of immunotherapy response. Tumor cells and their surrounding microenvironment can be shaped by different degrees of ferroptosis activation [29,30]. Through a series of signaling pathways [31,32], cancer cells going through ferroptosis have been shown to be able to find and activate immune cells in the TME [31,32]. Schreiber et al., co-founders of the Harvard Boulder Institute, found that an inhibition of glutathione peroxidase 4 (GPX4) induced ferroptosis in mesenchymal state resistant cancer cells [33]. Similar to GPX4-dependent mesenchymal resistant cancer cells, cancer cells with persistent drug resistance are also highly sensitive to ferroptosis, probably because they also exhibit mesenchymal characteristics [14]; therefore, studying the role of ferroptosis on the regulation of EMT will help us further understand the tumor microenvironment and explore the mechanisms of tumor drug resistance.

In this study, we combined the genomic information of gastric cancer samples from four different datasets and identified and comprehensively evaluated three different ferroptosis molecular clusters. We found that the TME characteristics of these three molecular clusters were highly consistent with the immune rejection phenotype, the immune inflammation phenotype, and the immune desert phenotype, which indicated that ferroptosis played a non-negligible role in shaping the characteristics of the individual tumor microenvironment. In addition, the ferroptosis molecular clusters were closely related to the immunotherapeutic response and prognosis of gastric cancer patients. To this end, we established a scoring system to quantify the ferroptosis molecular clusters of individual patients. The scoring system can help us target the tumor microenvironment through ferroptosis to enhance the effect of tumor immunotherapy, which may be a new cancer treatment strategy.

## 2. Results

### 2.1. Identification of Ferroptosis Molecular Subtypes and TME Characterization

**Figure 1:**
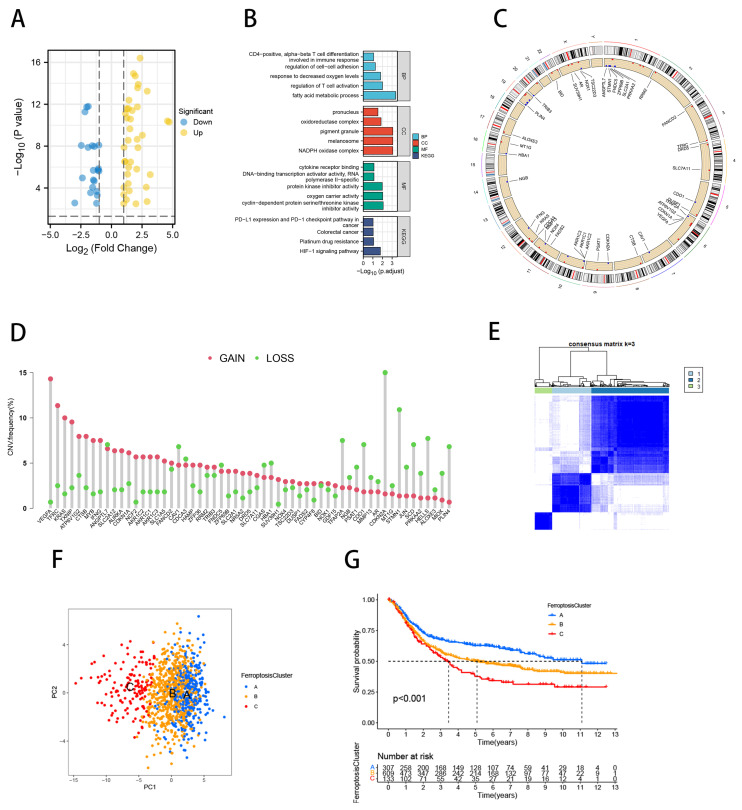
Identification of ferroptosis molecular subtypes. (**A**) Volcano plot demonstrating the differential expression of 59 ferroptosis-related genes in the TCGA-STAD cohort between tumors and adjacent non-tumor tissues. (**B**) Functional enrichment analysis of ferroptosis-related genes with differential expression. (**C**) CNV alteration sites of 59 ferroptosis-related genes. (**D**) CNV alterations of 59 ferroptosis-related genes. Column height indicates alteration frequency; green dots indicate deletions and red dots indicate amplifications. (**E**) The consensus cluster analysis divided the tumor samples into multiple subgroups, with the optimal K value = 3 (k is the number of subgroups divided). Clustering algorithm = K means method. (**F**) PCA of the mRNA expression profiles of FRGs from the GC samples confirmed the three clusters, A (blue), B (yellow) and C (red). (**G**) Kaplan–Meier survival curves for the three molecular clusters, A (blue), B (yellow) and C (red).

We identified 59 FRGs differentially expressed (FDR < 0.001, |log FC| > 1; Figure 1A, Table S1) between tumor and adjacent non-tumor tissues of the TCGA-STAD cohort by the “limma” package. In KEGG and GO analyses, these differentially expressed genes (DEGs) were enriched in regulating T cell differentiation, activation, regulating cell–cell adhesion, involved in the immune response, protein kinase inhibitor activity, cytokine receptor binding, hypoxia-inducible factor-1 signaling pathway, platinum resistance, and PD-L1 expression in tumors with the PD-1 checkpoint pathway (Figure 1B). We also examined the prevalence of somatic mutations and copy number variants (CNVs) of FRGs in GC. The mutation frequency of the FRGs of 433 samples was 34.18%, while KRAS was the most common mutation in the samples (Figure S1A). Figure 1C shows the location of CNV alterations in FRGs on the chromosome. The study of CNV alteration frequencies showed that CNV alterations were very common among the 59 regulatory factors and most of them were concentrated on copy number amplification (Figure 1D). The above analysis revealed that the genetics of GC are highly heterogeneous, which suggests that imbalances in the expression of FRGs are critical to the emergence and evolution of GC.

**Figure 2:**
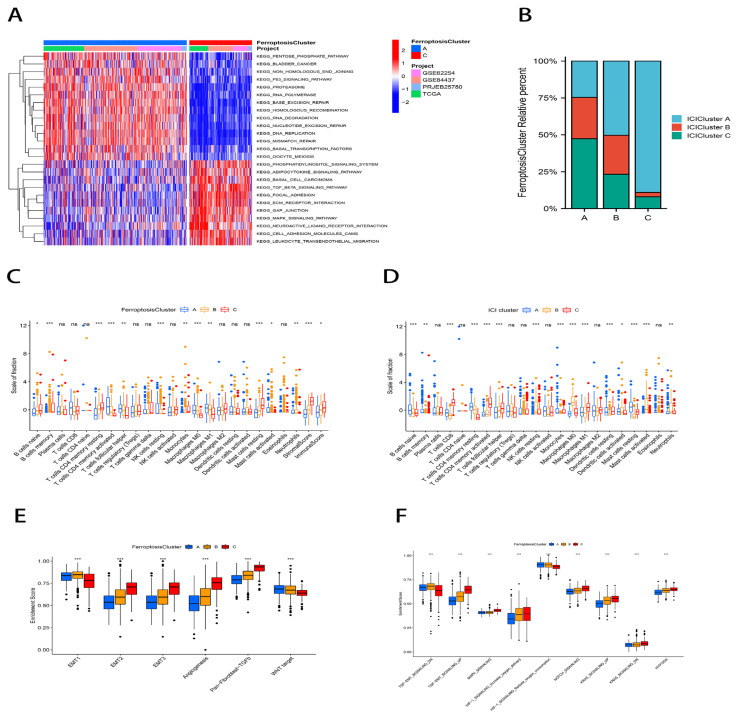
Tumor microenvironment characteristics of ferroptosis molecular subtypes. (**A**) GSVA analysis of distinct biological pathway activation in ferroptosis clusters A and C. Blue represents the repression pathway and red represents the activation pathway. (**B**) Percentage distribution of immune cell infiltration subtypes in each ferroptosis molecular subtype. (**C**,**D**) Box plots of immune infiltration levels in ferroptosis molecular subtypes (**C**) and immune cell infiltration subtypes (**D**). ns: not significant; * *p* < 0.05; ** *p* < 0.01; *** *p* < 0.001. (**E**,**F**) Differences in stromal activation pathway (**E**) and carcinogenesis-related pathways (**F**) in the three ferroptosis molecular subtypes. ns: not significant; * *p* < 0.05; ** *p* < 0.01; *** *p* < 0.001.

We used a consensus clustering approach to identify the molecular subtypes of ferroptosis. On the basis of the mRNA expression data of 59 FRGs, GC patients from four cohorts (TCGA-STAD, GSE84437, ACRG/GSE62254, and PRJEB25780) were classified into three molecular subtypes, namely, ferroptosis clusters A, B, and C (A: *n* = 329, B: *n* = 648, and C: *n* = 144; Figure 1E). The principal component analysis (PCA) confirmed that these three subtypes were fully distinguishable (Figure 1F). A prognostic analysis revealed a particular survival advantage for ferroptosis cluster A and the worst prognosis for ferroptosis cluster C (*p* < 0.001; Figure 1G). We conducted a gene set variation analysis (GSVA) enrichment analysis to investigate the biological behavior of the ferroptosis molecular subtypes (Table S2). As shown in Figure 2A, ferroptosis cluster A was significantly enriched in DNA repairing, basal transcription factor, and P53 signaling pathways. Cluster C was considerably abundant in stromal activation pathways including the TGF signaling pathway, adhesion plaque, ECM receptor interaction, MAPK signaling pathway, cell adhesion molecules (CAMs), and leukocyte transepithelial migration.

Afterwards, an unsupervised cluster analysis was conducted to classify the immune cell infiltration (ICI) subtypes of GC patients based on a cell-type identification by estimating the relative subsets of RNA transcript (CIBERSORT) calculations. As a result of the analysis of four cohorts of GC patients, three immune subtypes were identified, which are the ICI subtypes A, B, and C (A: *n* = 463, B: *n* = 221, and C: *n* = 259; Figure S1B). The degree of immune cell infiltration overlapping between ICI subtype C and ferroptosis cluster A was surprising to us. Figure 2B illustrates the distribution of each immune subtype in the ferroptosis clusters. A CIBERSORT analysis showed that ferroptosis cluster A exhibited elevated levels of immune cell infiltration, including T cells CD4 memory activated, NK cells resting, T cells follicular helper, macrophage M0, macrophage M1, and mast cells activated. The cluster C immune cell infiltration included B cells, monocytes, T cells, CD4 memory resting, and mast cells resting (Figure 2C). The ICI subtype C had significant immune cell infiltration, which was comprised of CD8+ T cells, T cells follicular helper, T cells CD4 memory activated, macrophage M1, and dendritic cells resting (Figure 2D). 

Similar to the ferroptosis molecular subtypes, the ICI subtype C had a particularly prominent survival advantage, and the ICI subtype A group had the worst prognosis (*p* = 0.04; Figure S1C). Despite having a significant immune cell infiltration, ferroptosis cluster C did not exhibit a corresponding survival benefit, according to our data. According to earlier research, tumors with an immune rejection phenotype also contain an abundance of immune cells, which remain within the peritumor stromal and do not exert an effective immune response [34]; therefore, we hypothesized that stromal activation of the ferroptosis cluster C inhibited the immune response against tumor cells. To this end, we performed an EMT analysis using the single sample Gene Set Enrichment Analysis (ssGSEA) method and showed significantly enhanced stromal activity in cluster C, such as an enrichment of the epithelial–mesenchymal transition (EMT), Pan-F-TBRS, and angiogenic pathways, which supported our hypothesis (Figure 2E). Further exploration of the relationship between the ferroptosis molecular subtypes and the regulatory mechanism of the EMT is shown in Figure 2F. We found that ferroptosis cluster C showed a significant enrichment in depleted oxygen-related signaling pathways as well as EMT-related signaling pathways (e.g., the TGF-β signaling pathway, MAPK signaling pathway, Notch signaling pathway, and KRAS signaling pathway).

### 2.2. Generation and Functional Annotation of Genomic Patterns Associated with Iron Deficiency

**Figure 3:**
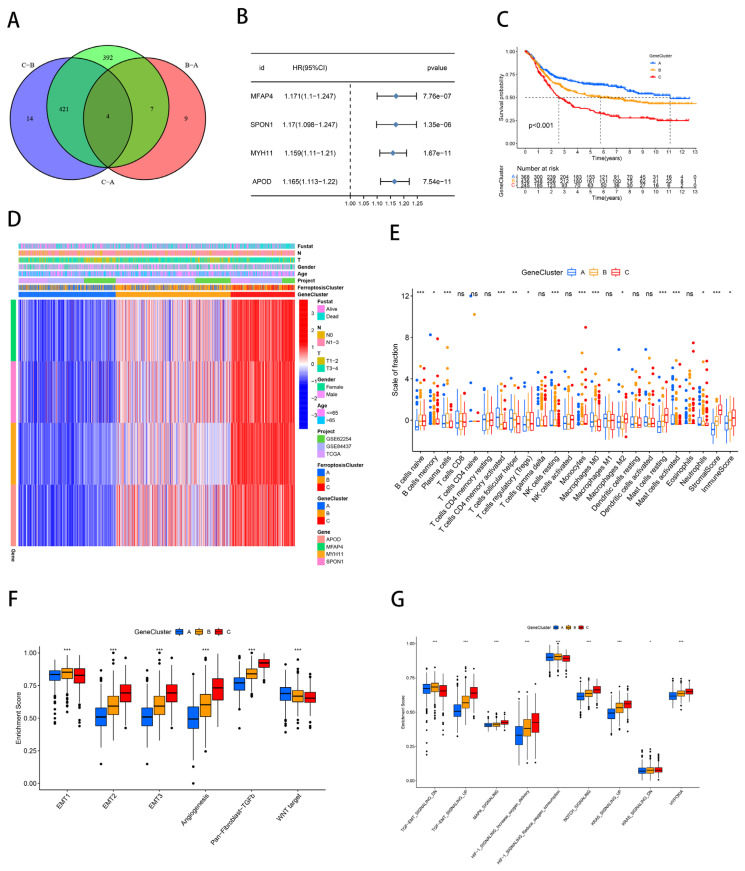
Generation and functional annotation of genomic patterns associated with iron deficiency. (**A**) Venn diagram showing the intersection of differentially expressed genes in the ferroptosis cluster. (**B**) Forest plot demonstrating univariate Cox analysis of genes associated with ferroptosis molecular subtypes. (**C**) Kaplan–Meier survival curves for the three gene clusters. (**D**) Heat map illustrating the relationship between the ferroptosis molecular subtype-associated gene expression, ferroptosis clusters, and various clinicopathological features. (**E**) Box plots depicting the levels of immune infiltration of the three genomic patterns. ns: not significant; * *p* < 0.05; ** *p* < 0.01; *** *p* < 0.001. (**F**,**G**) Differences in stromal activation pathway (**F**) and carcinogenesis-related pathways (**G**) in the three gene clusters. ns: not significant; * *p* < 0.05; ** *p* < 0.01; *** *p* < 0.001.

As a further investigation into the potential biological properties of ferroptosis molecular subtypes, we identified four DEGs (*APOD*, *MFAP4*, *MYH11*, and *SPON1*) associated with ferroptosis molecular subtypes using the “limma” package (FDR < 0.01, |log FC| > 1; Figure 3A). The Human Protein Atlas (HPA) database (https://www.proteinatlas.org/; accessed on 19 August 2022) was applied to compare the protein expression of these ferroptosis subtype-related DEGs in tumor and normal tissue (Figure S4) [35]. In a univariate Cox analysis, the four DEGs were strongly linked to the prognosis of patients with gastric cancer. (*p* < 0.001; Figure 3B). As expected, the GSEA analysis showed that the DEGs were enriched for many biological functions related to the chemokine signaling pathway, TGF-β signaling pathway, gap junctions, focal adhesion, cell adhesion molecule CAM, and ECM receptor interactions (Figure S2A–D).

A clustering analysis was conducted based on these DEGs in order to corroborate this regulatory mechanism in further detail. Patients from four cohorts were classified into three genomic patterns associated with ferroptosis, which we named gene clusters A, B, and C (A: *n* = 393, B: *n* = 465, and C: *n* = 263; Figure S2E). It was discovered that individuals in gene cluster A lived longer overall, while individuals in gene cluster C had gloomy outlooks (*p* < 0.001; Figure 3C). The heat map showed that the gene clusters were similar to the ferroptosis molecular subtypes. And the expression of four DEGs (*APOD*, *MFAP4*, *MYH11*, and *SPON1*) associated with the ferroptosis molecular subtypes was significantly up-regulated in gene cluster C (Figure 3D). The genomic pattern of immune infiltration, EMT, and other biological signaling pathways were explored using the ssGSEA analysis, and the findings also verified that gene cluster C was typified by the same stromal activation and tumor-promoting state as ferroptosis cluster C (Figure 3E–G). Figure S2F stated the distribution of each immune subtype in the gene clusters. In conclusion, the genomic pattern of ferroptosis was consistent with the expected outcome of the molecular subtypes of ferroptosis in terms of the immune infiltration, stromal activation, and survival.

### 2.3. Development of an Independent Prognostic Model for Gastric Cancer Based on Ferroptosis

**Figure 4:**
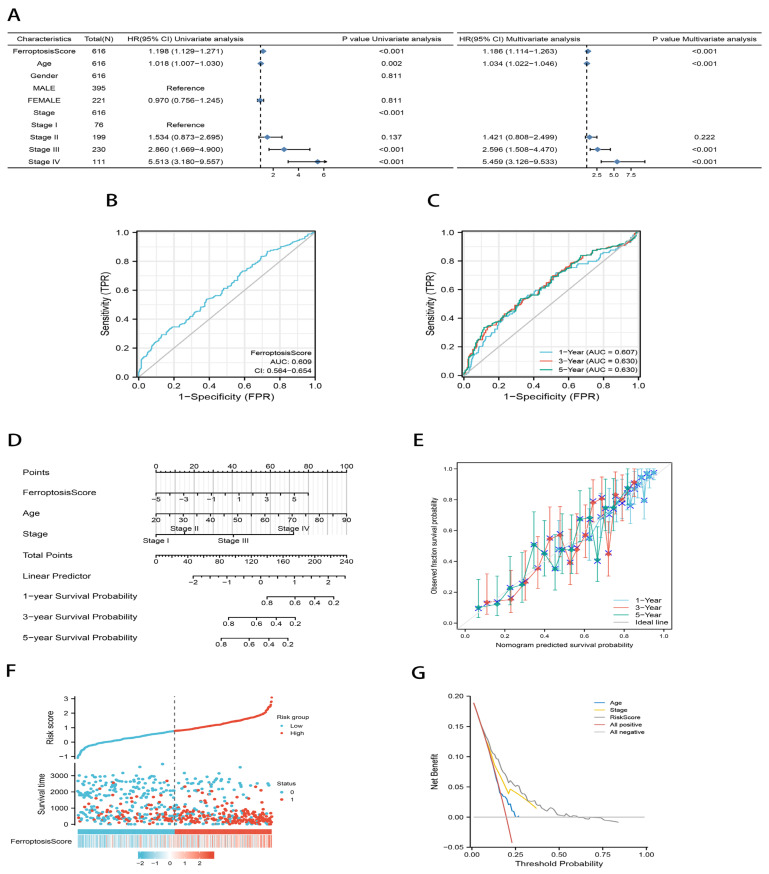
Development of an independent prognostic model for gastric cancer based on ferroptosis. (**A**) Univariate and multivariate Cox analysis of ferroptosis score. (**B**,**C**) Diagnostic ROC curve (**B**) and time-dependent ROC curve (**C**) for ferroptosis score. AUC: area under the curve. (**D**) A nomogram plot of prognostic multivariate regression model. (**E**) Prognostic calibration plot evaluating the fit analysis of the model to the actual situation. (**F**) Risk factor plot showing the distribution of survival outcomes using the median risk score of the prediction model for grouping patients with gastric cancer. (**G**) Prognostic DCA plot evaluating the clinical utility of the prognostic model.

The aforementioned research based solely on the patient group is unable to effectively forecast the pattern of ferroptosis in specific patients due to the variety and complexity of individual gastric cancers. We used a principal component analysis (PCA) to quantify the ferroptosis molecular subtypes of individual tumors and constructed a scoring system, which we refer to as the ferroptosis score [36,37]. We first removed samples with missing clinical data and finally included 616 samples. The ferroptosis score was confirmed as an independent prognostic factor for gastric cancer by a univariate and multivariate Cox analysis (*p* < 0.001; Figure 4A). Sensitivity (TRP) in the diagnostic receiver operating characteristic (ROC) curve and time-dependent ROC curve represents the true positive rate (sensitivity), and 1-Sensitivity (FRP) represents the false positive rate (specificity). The area under the curve (AUC) of the ROC was all greater than 0.5, suggesting that the ferroptosis score had good sensitivity and specificity (Figure 4B,C). Next, a nomogram plot was used to analyze and visualize the prognostic multifactorial regression model for the scenario. The ferroptosis score and other independent clinical risk factors (age and stage) were evaluated according to their survival risk. Compared with other clinical characteristics, the ferroptosis score contributed risk points from 0 to 80, second only to age (Figure 4D). The calibration curves for the overall survival (OS) are shown in Figure 4E. The comparison lines between the 1-year survival, 3-year survival, and 5-year survival predicted by the model and the actual situation were close to the diagonal, suggesting that the ferroptosis scoring model had a good fitting effect. Compared with the ROC curve, only sensitivity and specificity can be used to evaluate the quality of the model, and only the accuracy is considered. The prognostic decision curve analysis (DCA) diagram considers the clinical utility or patient benefit of the model. When it comes to the patient benefit, the multivariate prognostic model made by combining the ferroptosis scores is better than either age or stage alone (Figure 4G). 

### 2.4. Correlation Study of Ferroptosis Scores and Clinical Characteristics in GC Patients

**Figure 5:**
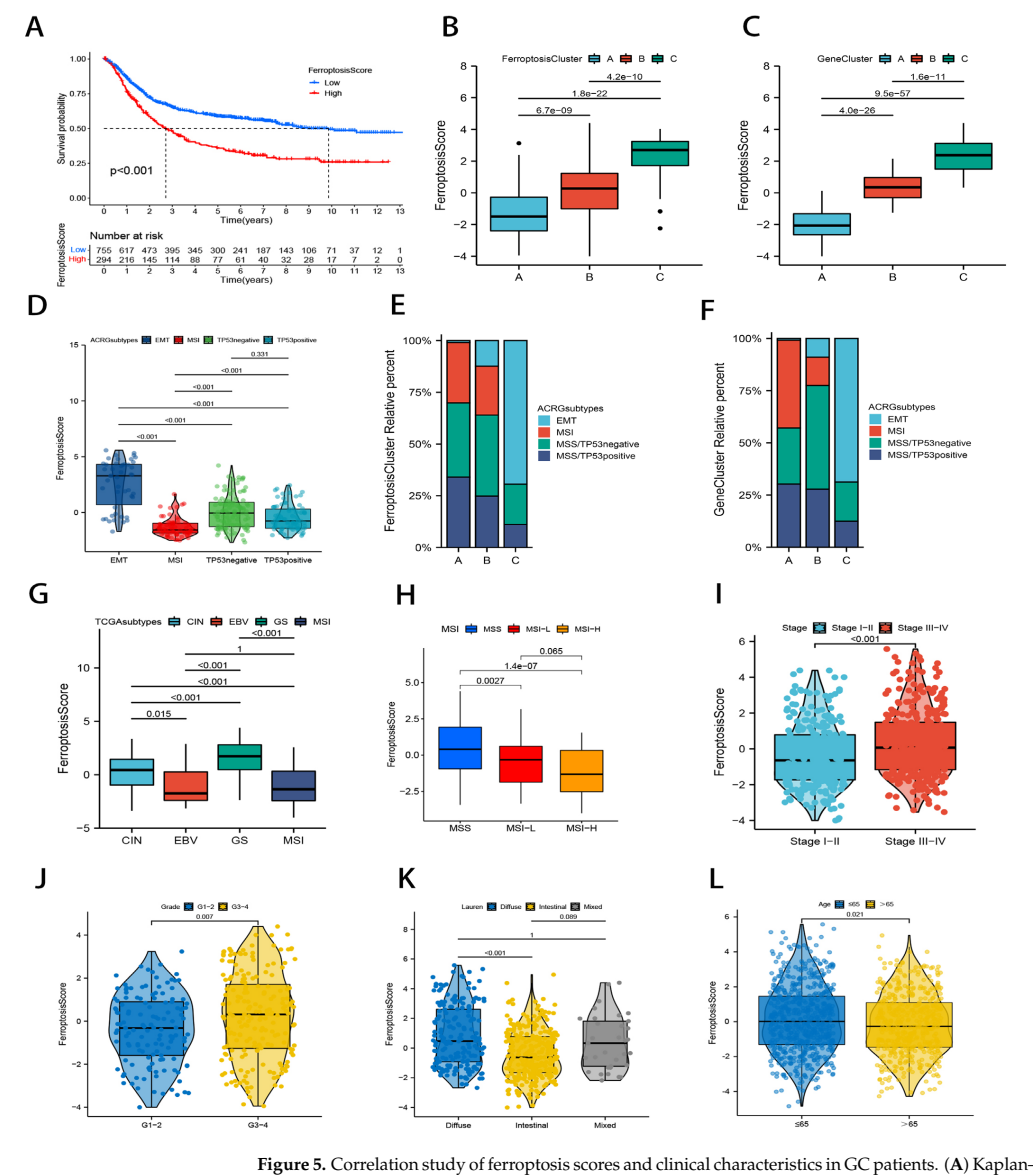
Correlation study of ferroptosis scores and clinical characteristics in GC patients. (**A**) Kaplan–Meier survival curves for the ferroptosis score group. (**B**,**C**) Differences in ferroptosis scores in the ferroptosis cluster (**B**) and gene cluster (**C**). (**D**) Differences in ferroptosis scores between ACRG types. (**E**,**F**) ACRG type distribution in ferroptosis clusters (**E**) and gene clusters (**F**). (**G**) Differences in ferroptosis scores for TCGA types. (**H**–**L**) Relationship between ferroptosis scores and clinical features such as MSI (**H**), stage (**I**), grade (**J**), Lauren pathological classification (**K**), and age (**L**).

To continue, we sought to further determine the correlation between ferroptosis scores and clinical features of gastric cancer. According to the ideal cutoff (1.131985), patients from the three cohorts (TCGA-STAD, GSE84437, and ACRG/GSE62254) were separated into high and low ferroptosis score groups (high group: *n* = 294, and low group: *n* = 755). Consistent with our previous study, patients with low ferroptosis scores had a considerably better outcome than those with high ferroptosis scores, according to Kaplan–Meier curves (*p* < 0.001; Figure 5A). The Kruskal–Wallis test revealed that the ferroptosis scores in the ferroptosis clusters were significantly different, as were the gene clusters (*p* < 0.01; Figure 5B,C). Consistent with the predicted results, the ferroptosis cluster C patients had the poorest prognosis and the highest ferroptosis score, whereas the class A patients displayed the opposite characteristics. Not surprisingly, Figure 5C shows that the gene clusters showed the same results in the ferroptosis score.

Then, using the Kruskal–Wallis test method in the ACRG cohort of GC patients, we looked into the correlation between the ferroptosis scores and ACRG typing. Figure 5D shows that the ferroptosis scores were significantly different in all ACRG types except for MSS/TP53- and MSS/TP53+ (*p* < 0.001). It has been demonstrated that the diffuse histological type and EMT subtype are substantially linked to a worse prognosis in cases of gastric cancer [38]. Microsatellite instability (MSI), which accompanies DNA mismatch repair defects, is a crucial clinical tumor indicator and one of the most common predictors of efficacy in immunotherapy [38,39]; therefore, MSI subtypes were associated with better clinical outcomes. Most EMT subtype patients in our study clustered in ferroptosis cluster C and gene cluster C, whereas hardly any EMT subtype patients clustered in cluster A. This finding further highlighted the important relationship between stromal activation and ferroptosis cluster C (Figure 5E,F). The TCGA study classified primary gastric cancer into four subtypes based on the clustering results: microsatellite instability (MSI), EB virus infection (EBV), chromosomal instability (CIN), and genomic stability (GS) [3]. Among the four subtypes, GS type gastric cancer has the worst prognosis, while the EBV subtype has the best prognosis. Although the ferroptosis scores did not differ significantly between the EBV and MSI subtypes in TCGA, patients with the GS subtype had the highest ferroptosis scores and those with the EBV subtype had the lowest ferroptosis scores, which was consistent with our expected results (Figure 5G). We also explored the connections between the ferroptosis scores and other clinical traits in GC patients using the Wilcoxon rank sum test and Kruskal–Wallis test method. A significant difference was found in the ferroptosis scores according to MSI, staging, grading, Lauren pathology, and age (Figure 5H–L). The clinical features associated with a good prognosis all had relatively low ferroptosis scores.

### 2.5. Ferroptosis Score for Tumor Somatic Cell Mutation Characterization

**Figure 6:**
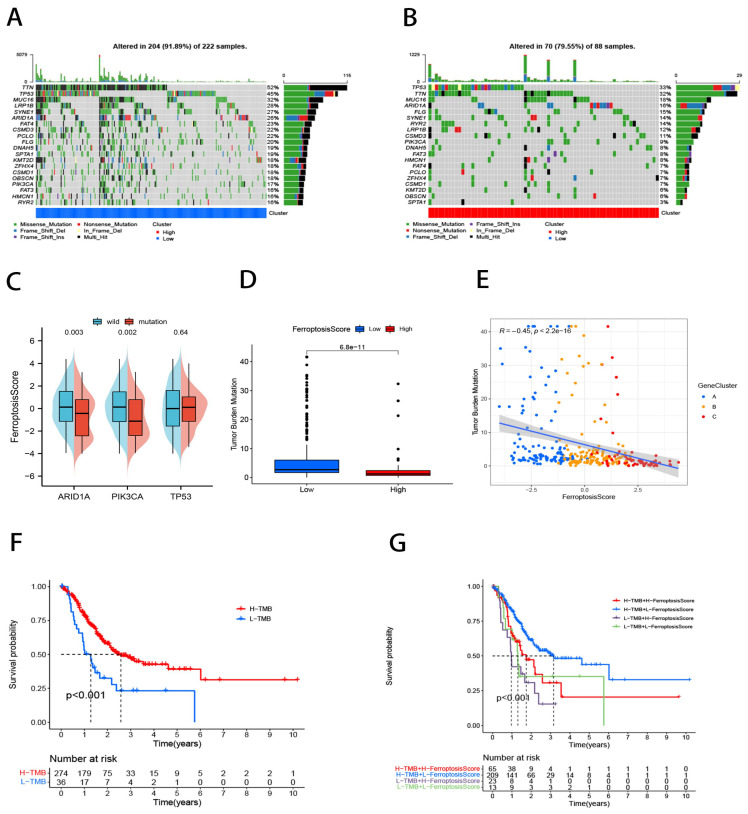
Tumor somatic cell mutation characteristics of ferroptosis score. (**A**,**B**) Waterfall plots of tumor somatic mutations in patients with low (**A**) and high (**B**) ferroptosis scores. Each column represents one patient. The top bar indicates the degree of tumor mutation. The numbers on the right indicate the frequency of mutations in each gene. The bars on the right show the proportion of different types of mutations. Stacked bar graphs show the conversion rate for each sample. (**C**) Ferroptosis scores differ between wild-type and mutant *ARID1A*, *PIK3CA*, and *TP53*. (**D**) Box diagram illustrating the difference in tumor mutation burden between ferroptosis score groups. (**E**) Spearman’s Rank Correlation of ferroptosis score, tumor mutation burden, and gene clusters. (**F**) Kaplan–Meier curve for tumor mutation burden groupings. (**G**) Kaplan–Meier curve for ferroptosis and tumor mutation burden.

Based on our data, we discovered that gene mutations differed significantly between those with high and low ferroptosis scores (Figure 6A,B). We applied a Fisher’s test and found 136 genes exhibiting significant mutational differences across the ferroptosis score groups (*p* < 0.05; Table S3). Additionally, we explored TCGA-TSAD in specific altered genes and the ferroptosis scores for the mutant *ARID1A* and *PIK3CA* phenotypes were markedly lower than those for the wild-type (Figure 6C). Patients with low ferroptosis scores had a relatively higher TMB (Figure 6D,E). In GC patients, a high TMB has been shown to lead to improved outcomes [40], which is in agreement with our findings (Figure 6F,G).

### 2.6. Ferroptosis Scores Correlated with TME Characteristics

**Figure 7:**
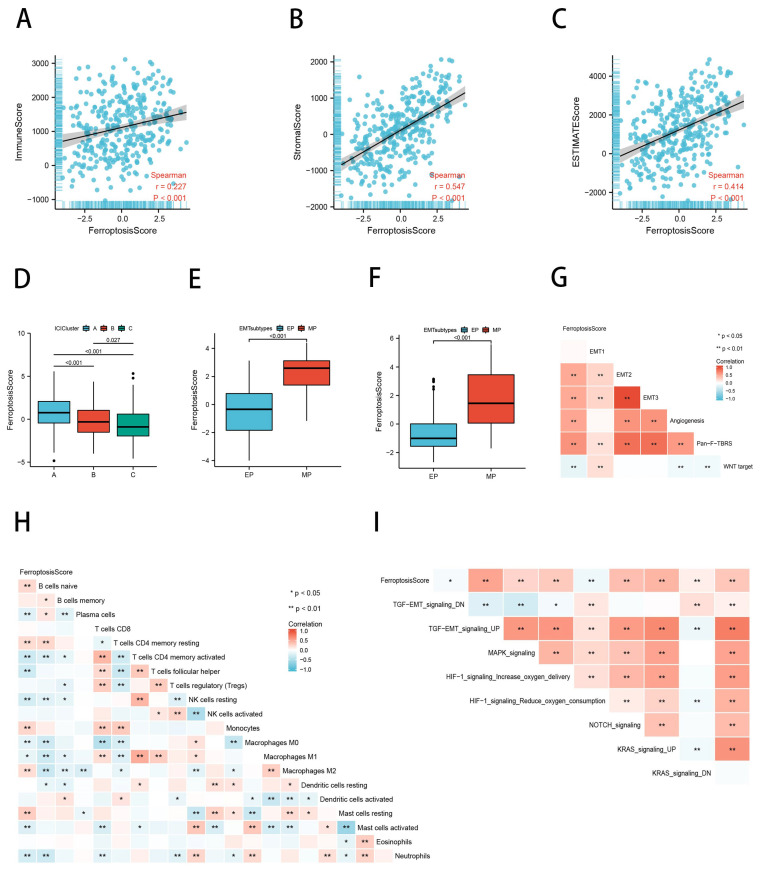
Ferroptosis scores correlate with TME characteristics. (**A**–**C**) Spearman’s Rank Correlation analysis between ferroptosis score and immune score (**A**), stromal score (**B**) and ESTIMATE score (**C**). (**D**) Ferroptosis scores differ between ICI subtypes. (**E**,**F**) Differences in ferroptosis scores for EMT typing in the TCGA cohort (**E**) and the ACRG cohort (**F**). (**G**–**I**) Heat map of the correlation between ferroptosis score and stromal activity (**G**), immune cell infiltration (**H**), and EMT-related signaling pathways (**I**).

The tumor microenvironment consists primarily of resident stromal cells and immune cells that recruit from the surrounding tissues; therefore, in order to facilitate the assessment of immune as well as stromal elements of the TME, we calculated the immune score, stromal score, and Estimation of Stromal and Immune Cells (ESTIMATE) score of GC samples using the ESTIMATE. The results of the Spearman’s Rank Correlation analysis revealed a positive association between the ferroptosis score and the ESTIMATE score, immune score, and stromal score (Figure 7A–C). We subsequently found statistically significant differences in the ferroptosis scores between the immune subtypes, with the ICI subtype A having the highest score (Figure 7D). In 2018, Oh et al. examined genomic and proteomic data to identify two distinct types of gastric cancer: the mesenchymal phenotype (MP) and epithelial phenotype (EP) [41]. The survival and chemosensitivity of these two subtypes differed significantly; therefore, we analyzed the difference in ferroptosis scores between the EP and MP subtypes in the ACRG cohort. According to the findings, the MP subtype ferroptosis scores in the TCGA cohort were statistically higher than the EP subtype (*p* < 0.001; Figure 7E). This was confirmed in the ACRG cohort as well (*p* < 0.001; Figure 7F). Not surprisingly, in the subsequent analysis at ssGSEA, the ferroptosis scores showed a significant positive correlation with tumor stromal activation (Figure 7G,I). In order to determine if there was an association between the ferroptosis score and immune cell infiltration, we performed a Spearman’s Rank Correlation analysis (Figure 7H). The ferroptosis score was found to be positively correlated with macrophage M2 infiltration and negatively correlated with T cell CD4 memory activated, T cell follicular helper, macrophages M1, mast cells activated, and neutrophil infiltration. These data suggest that ferroptosis may be involved in regulating the tumor microenvironment, thereby influencing tumor growth and progression.

### 2.7. The Ferroptosis Score Correlated with Immunotherapy Efficacy and Chemotherapy Drug Sensitivity Prediction

**Figure 8:**
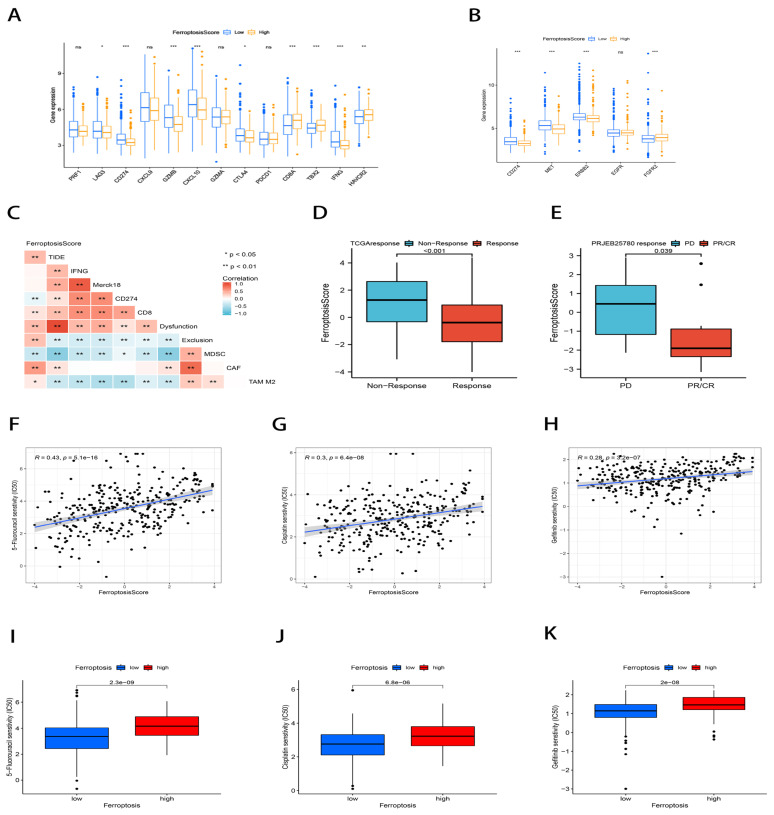
Ferroptosis score correlated with immunotherapy efficacy and chemotherapy drug sensitivity prediction. (**A**,**B**) Box plots depicting the relative expression differences of checkpoints (**A**) and common targets of gastric cancer (**B**) between ferroptosis score groups. ns: not significant; * *p* < 0.05; ** *p* < 0.01; *** *p* < 0.001. (**C**) Correlation heat map demonstrating the association of ferroptosis scores with TIDE scores. (**D**,**E**) Differences in ferroptosis scores between the ICB treatment response and non-response groups in the TCGA cohort (**D**) and PRJEB25780 cohort (**E**). (**F**–**H**) Correlation of ferroptosis scores with chemotherapeutic drug sensitivity, including 5-fluorouracil (**F**), Cisplatin (**G**), Gefitinib (**H**). **(I**–**K)** Differences in chemotherapy drug sensitivity between ferroptosis score subgroups, including 5-fluorouracil (**I**), Cisplatin (**J**), and Gefitinib (**K**).

To assess the immunological response and tolerance to immunotherapy in GC patients. As the immune checkpoint-related signatures, we chose *CD274*, *CTLA4*, *LAG3*, *HAVCR2*, *IDO1*, and *PDCD1*; as the immunological activity-related signatures, we chose *CD8A*, *CXCL10*, *GZMA*, *CXCL9*, *GZMB*, *GZMA*, *IFNG*, *PRF1*, *TBX2*, and *TNF* [42,43]. In the group with low ferroptosis scores, the majority of immunological checkpoint-associated and immunoreactive-related markers were found to be significantly overexpressed (Figure 8A). Additionally, we investigated the connection between the ferroptosis score and the gene expression of popular GC medication targets and discovered that the majority of the target genes were considerably overexpressed in the group with low ferroptosis scores (Figure 8B).

In light of the significance of immunotherapy in cancer treatment, we further examined the connection between the ferroptosis score and immune checkpoint blockade (ICB) response using clinical data from TCGA-STAD and PRJEB25780. The Tumor Immune Dysfunction and Exclusion (TIDE) algorithm was used to evaluate patients in the TCGA-STAD cohort, and it was discovered that the ferroptosis score had a positive correlation with the TIDE score by using Spearman’s Rank Correlation (*p* < 0.01; Figure 8C), whereas the ferroptosis score was significantly lower in the ICB treatment response group than in the non-response group (*p* < 0.01; Figure 8D). This finding was similarly validated in the PRJEB25780 cohort (*p* = 0.039; Figure 8E). Taken together, this evidence strongly supports the predictive efficacy of the ferroptosis score on immunotherapy outcomes.

Studies have shown that sorafenib, as a classical targeted chemotherapy drug, has been shown to exert cytotoxic effects on hepatocellular carcinoma cells through ferroptosis [44,45]. A study has also shown that Cisplatin, a classical therapeutic drug, can induce ferroptosis in A549 and HCT116 cells and shows significant synergistic antitumor activity when combined with Cisplatin and erastin [46]. We examined the connection between the ferroptosis score and the half maximum inhibitory concentration (IC_50_) of chemotherapeutic medicines. Numerous medications, including 5-fluorouracil, Cisplatin, Gefitinib, and many others, significantly correlated with the ferroptosis score. The high ferroptosis score group reported greater IC_50_ estimates when compared to the low ferroptosis score group (Figure 8F–K). In conclusion, a high ferroptosis score predicted an increased sensitivity to these therapeutic agents in GC patients. The ferroptosis scoring system can stratify patients, screen sensitive patients, and find a new way to overcome the related chemoresistance problems.

### 2.8. Pan-Cancer Analysis of Ferroptosis Scores

**Figure 9:**
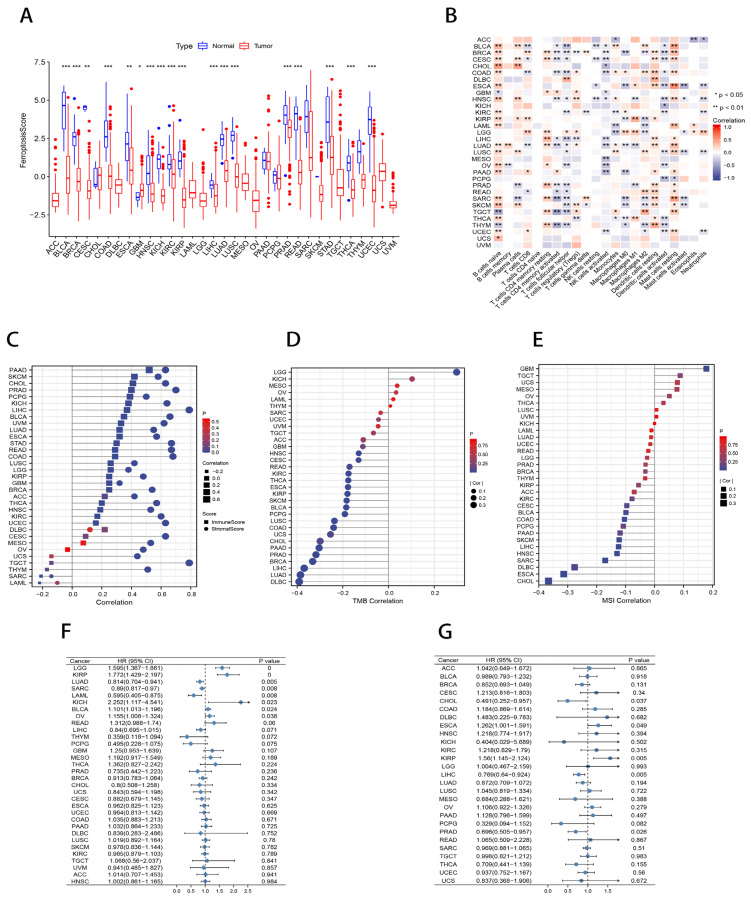
Pan-cancer analysis of the ferroptosis score. (**A**) Differential expression analysis of the ferroptosis score between tumor tissues and adjacent normal tissues. * *p* < 0.05; ** *p* < 0.01; *** *p* < 0.001. (**B**) Pan-cancer landscape associated with ferroptosis score and immune cell infiltration. * *p* < 0.05; ** *p* < 0.01. (**C**) Lollipop charts describing the correlation of the ferroptosis score in pan-cancer with the ESTIMATE score. (**D**,**E**) Lollipop charts of the Spearman’s Rank Correlation between ferroptosis score and TMB (**D**) and MSI (**E**) in pan-cancer. (**F**,**G**) Univariate Cox analysis of ferroptosis score when the clinical outcome was OS (**F**) and DFS (**G**).

The differential expression analysis between tumor tissues and normal tissues adjacent to cancer showed that there were significant differences in the ferroptosis scores among 18 cancers (*p* < 0.05; Figure 9A). To comprehensively describe the immunological characteristics of ferroptosis and the clinical correlation with multiple malignancies, we examined ferroptosis scores in 32 cancers and performed a Spearman’s Rank Correlation analysis. Figure 9B illustrates the correlation between the ferroptosis score and immune cell infiltration in the pan-cancer landscape. We found that the ferroptosis score was associated with immune cells in most cancer types. The ferroptosis score was also strongly connected with the stromal score and immune score according to the ESTIMATE (Figure 9C). In addition, the Spearman’s Rank Correlation analysis between the ferroptosis scores and tumor mutational burden (TMB) in 32 cancer types showed that in 1 cancer, the ferroptosis scores were positively related to TMB, whereas in 17 cancers, they were negatively related to the TMB (*p* < 0.05; Figure 9D). Ferroptosis scores were connected with MSI in one cancer in a positive way and in eight cancers in a negative way (*p* < 0.05; Figure 9E). Forest plots of the univariate Cox analysis show that different ferroptosis scores predicted survival in eight cancer types when the clinical outcome was overall survival (OS) (*p* < 0.05; Figure 9F); however, when the clinical outcome was disease-free survival (DFS), the ferroptosis scores were predictive in only five cancers (*p* < 0.05; Figure 9G). The Kaplan–Meier survival curves show the survival difference of the ferroptosis score when the clinical outcome was OS, DFS, disease-specific survival (DSS), or progression-free survival (PFS) (*p* < 0.05; Figure S3). The Pan-cancer analysis results are presented in Supplementary Table S6.

## 3. Discussion

With major advances in ICB monotherapy for progressive solid tumors in recent years, clinical investigators have conducted numerous explorations in gastric cancer, ranging from ATTRACTION-2 clinical studies after the failure of standard chemotherapy to KEYNOTE-062 and CheckMate-649 s- and first-line clinical trials [4,5,6,7,47]. Despite the bumps along the way, an important breakthrough has been made that offers hope for the treatment of advanced gastric cancer patients; however, despite their impressive efficacy, treatment failure, drug resistance, and recurrence remain common [48,49]. Numerous studies have linked ferroptosis to the death of tumorigenic cells such as hepatocellular carcinoma, glioma, non-small cell lung cancer, and breast cancer [14,50,51,52,53]. As a result, targeting ferroptosis could be a novel approach to cancer treatment [54]; however, there are still relatively few studies on the role of ferroptosis in gastric cancer, especially the potential regulatory mechanisms in relation to TME characteristics, immunotherapy response, and prognosis, which remain to be further investigated.

In this current investigation, 59 FRGs that displayed differential expression between GC tumor tissues and surrounding non-tumor tissues were examined. These FRGs were crucial for the malignant tumorigenesis, proliferation, metastasis, and even drug resistance of malignant tumors; therefore, we identified three ferroptosis molecular subtypes based on the mRNA expression profiles of the FRGs. In terms of prognosis, molecular function, immune infiltration microenvironment, and immunotherapeutic response, these three subtypes varied significantly. The immune cell infiltration and EMT assessment by CIBERSORT revealed that cluster A was distinguished by significant immune activation and immune cell infiltration, cluster B by immune deficiency, and cluster C by immunosuppression and tumor stromal activity. To verify the TME characteristics of the ferroptosis subtypes, we also established ICI subtypes (e.g., immune-infiltrated phenotype, immune-desert phenotype, and immune-excluded phenotype) by the results of the CIBERSORT arithmetic. The results showed that the ICI subtypes and ferroptosis subtypes have a high overlap. Hegde classified the tumor microenvironment into three types in 2016 [55]. Among them, the immunoinflammatory phenotype is characterized by tumor infiltration with a high number of immune cells, such as T cells, and high levels of PD-L1 expression in tumor cells. Numerous basic and clinical studies have shown that immunotherapy can significantly benefit inflammatory tumors [34,55]. The immune-excluded phenotype, on the other hand, is distinguished by the existence of significant immunosuppressive cells in the tumor microenvironment, activation of the TGF-β signaling pathway, increased infiltration of myeloid inflammatory cells, and significant tumor vascular proliferation. Consistent with the above definition, we found that the ICI subtype A patients with the best prognosis were significantly infiltrated with more CD8+ T cells and macrophage M1. Data from the immunotherapy cohort of uroepithelial carcinoma demonstrated that patients benefited more from checkpoint inhibitors with higher levels of infiltration of macrophage M1 [28]. This is similar to the ferroptosis molecular subtypes, suggesting that CD8+ T cell and macrophage M1 infiltration underlie the microenvironment in which patients benefit from immunotherapy. In contrast, cluster C exhibited a significant state of stromal activation, including a high expression of EMT associated signaling pathways (e.g., the TGF-β signaling pathway, Notch signaling pathway, MAPK signaling pathway, and KRAS signaling pathway), activation of Pan-F-TBRS, and angiogenic pathways, which are thought to inhibit T cells [28,56]; thus, it is not surprising that cluster C innate immunity is activated but has a poorer prognosis.

Transcriptome differential genes between the different ferroptosis molecular subtypes showed enrichment for biological processes significantly associated with stromal components and immune activation. As a result, these genes that were expressed differently were regarded as ferroptosis-associated signature genes. Unsupervised cluster analysis based on these ferroptosis-related signature genes identified three gene clusters. Moreover, the genomic subtypes had almost the same prognostic and TME characteristics as the ferroptosis molecular subtypes. These results suggest that different ferroptosis molecular subtypes do exist in GC. Our comprehension of the cellular infiltration properties of TME would be improved by a thorough analysis of the ferroptosis molecular subtypes. In order to account for the individual heterogeneity of GC, we established a ferroptosis score to assess the molecular subtypes of ferroptosis in specific GC patients and separated GC patients into high and low ferroptosis score groups. Ferroptosis molecular subtypes characterized by an immune rejection phenotype had higher ferroptosis scores, whereas ferroptosis molecular patterns characterized by an immune inflammatory phenotype had lower ferroptosis scores.

As expected, the survival analysis showed that individuals in the low ferroptosis score group experienced better survival expectations. The diagnostic ROC curve and time-dependent ROC curve also validated the sensitivity and specificity of the ferroptosis score in predicting the survival outcomes in GC. We developed a prognostic multifactorial regression model incorporating the ferroptosis score and other independent clinical risk factors (age and stage). We verified that the ferroptosis score has a good predictive efficacy on the outcome of gastric cancer patients and confirmed that the ferroptosis score is a trustworthy indicator for patient survival assessment from various aspects. Further investigation of the relationship between the ferroptosis score and clinical characteristics of GC patients found that a high ferroptosis score was significantly associated with most clinical features of a poor GC prognosis (e.g., MSS, advanced stage, diffuse type, advanced age, and low PD-L1 expression). Moreover, ferroptosis cluster C was the predominant component of the EMT subtype in the ACRG cohort, which again confirmed the ferroptosis cluster C stromal activation feature. According to the aforementioned analysis, stromal activity, a high malignancy, and rapid advancement were substantially related to tumors with high ferroptosis scores.

The current state of research suggests that the formation of an immunoinflammatory tumor microenvironment stems primarily from genomic variants in the tumor, such as MSI and TMB, that increase the production of tumor neoantigens, resulting in a tumor microenvironment infiltrated with large numbers of TILs that can attack tumor cells [39,57,58,59]. The ferroptosis score and TMB had a very strong negative association according to the correlation analysis [39,57,58]; thus, the above results indirectly suggest the potential of ferroptosis scores to predict the responsiveness to immunotherapy.

According to the Spearman’s Rank Correlation analysis, the ESTIMATE score, immune score, and stromal score all had favorable correlations with the ferroptosis score. These findings suggest that ferroptosis is related to the ratio of immune and stromal components. Furthermore, we looked into the molecular underpinnings of EMT. The scores for ferroptosis demonstrated a substantial positive connection with stromal activity and signaling pathways connected to EMT. Previous research has shown that activating the EMT and TGF-β related pathways decreases the transport of T cells into tumors and their ability to destroy malignancies [28,56]. According to earlier theories, activated stromal TME may impact the accuracy of gastric cancer immunotherapy and induce therapeutic resistance to immune checkpoint inhibition. The aforementioned findings, therefore, infer that variations in tumor ferroptosis have a non-negligible role in forming various stromal and immunological TME landscapes and may be a significant component in modulating the clinical efficacy of ICB therapy. Consequently, we validated the predictive effect of the ferroptosis scores on the immunotherapy response in the TCGA-STAD and PRJEB25780 cohorts. It was observed that patients tended to respond better to immunotherapy when their ferroptosis scores were lower. The half maximum inhibitory concentration (IC_50_) of several medications, including 5-fluorouracil, Cisplatin, and Gefitinib, revealed a significant positive connection with the ferroptosis score, according to our analysis of drug sensitivity. In conclusion, high ferroptosis scores predicted an increased sensitivity to these therapeutic agents in GC patients. In a pan-cancer analysis, we examined the relationships between the ferroptosis scores and MSI, TMB, survival prognosis, and ESTIMATE scores in 32 cancers. It has been shown that the ferroptosis gene signature combines several factors, including stromal and immunological TME, mutational burden, MSI condition, and PD-L1 expression, and may be a more accurate immunotherapy prediction method.

## 4. Materials and Methods

### 4.1. Data Collection

Publicly available gastric cancer transcriptomes and corresponding clinical annotations were collected from The Cancer Genome Atlas (TCGA, https://portal.gdc.cancer.gov/repository; accessed on 20 February 2022) database. For validation, 733 gastric cancer patients were acquired from two datasets, GSE84437 and ACRG/GSE62254, from the Gene Expression Compendium (GEO) database (https://www.ncbi.nlm.nih.gov/geo; accessed on 12 February 2022). The anti-PD-1 treatment cohort PRJEB25780 with clinical and standardized RNA expression data was obtained from the Tumor Immune Dysfunction and Exclusion database (TIDE, http://tide.dfci.harvard.edu/; accessed on 20 March 2022) [60]. We collected clinical data from the manuscript [61]. The TCGA-STAD copy number variant (CNV) data was extracted from the UCSC xena (https://xena.ucsc.edu/; accessed on 20 February 2022) database. We used the ComBat method to remove the batch effect of the gene expression data.

A list of ferroptosis-related genes (FRGs) was obtained from the FerrDb website (http://www.zhounan.org/ferrdb; accessed on 21 March 2022), which is the first database on ferroptosis regulators and indicators and associations with disease [62]. In addition, we removed duplicate genes, leaving us with 380 genes for further investigation (Table S4). All data from TCGA, GEO, TIDE, and FerrDb is publicly available. Data access policies and release guidelines for TCGA and GEO were strictly adhered to in this study.

### 4.2. Construction of Ferroptosis Molecular Subtypes in the TCGA-STAD Cohort

The RNA sequencing data (FPKM values) from the TCGA-STAD dataset were converted to TPM using R (version 4.0.3) and the R package “TCGAbiolinks” [63]. The R “limma” package was used to analyze and screen gastric cancer samples and paracancerous tissues from the TCGA database for differentially expressed FRGs (FDR < 0.01, |logFC| > 1) [64].

We performed unsupervised clustering analysis using the “ConsensuClusterPlus” to identify the molecule subtypes of ferroptosis according to the mRNA expression data of differentially expressed FRGs [65]. The number of clusters and their stability were calculated using the consensus clustering approach [66]. In order to ensure the stability of the classification, the analysis was repeated 1000 times; therefore, patients from the TCGA-STAD, GSE62254, GSE84437, and PRJEB25780 cohorts were classified for further analysis.

### 4.3. Functional Enrichment Analysis and Immune Microenvironment (TME) Characterization

Gene ontology (GO) and Kyoto Encyclopedia of Genes and Genomes (KEGG) analyses of differential genes (FDR < 0.05) were performed utilizing the “clusterProfiler” [67]. In order to investigate potential differences in the biological processes between the ferroptosis clusters, we downloaded the gene set “c2.cp.kegg.v7.4” from the MSigDB database and completed a gene set variation analysis (GSVA) through the “GSVA” R package. GSVA is a commonly used, non-parametric, unsupervised method for estimating biological pathways and processing variation in samples of expression datasets [68]. The difference between the two groups was assessed as statistically significant when the corrected *p* < 0.05 was reached. To investigate the stromal state in the tumor microenvironment, we obtained a set of genes associated with multiple biological processes from Mariathasan et al., including epithelial–mesenchymal transition (EMT) marker gene sets EMT1, EMT2, and EMT3; angiogenic signature; TGF-β response signature of pan-fibroblasts (Pan-FTBRS); WNT targets [28]. To further reveal the mechanisms by which ferroptosis affects the tumor immune microenvironment, we downloaded EMT-related gene sets from the MSigDB database [69], including: TGF-EMT signaling down; TGF-EMT signaling up; MAPK signaling; NOTCH signaling; KRAS signaling up; KRAS signaling down; hallmark-hypoxia; HIF-1 signaling to increase oxygen delivery; HIF-1 signaling to decrease oxygen consumption [20,70]. The relevant signaling pathway information is presented in Supplementary Table S5. The mechanism of the TME signature generation was then explored through a Single Sample Gene Set Enrichment Analysis (ssGSEA) [42]. Estimation of Stromal and Immune Cells (ESTIMATE) implements an estimation algorithm through gene expression profiling, generating scores to calculate the level of infiltrating stromal cells, immune cells, and tumor purity in malignant tissues [71].

CIBERSORT from the Alizadeh lab is an analytical tool developed by Newman et al. It was first published in *Nature Methods* in 2015 and has since become one of the most widely used tools for quantifying and analyzing immune cell infiltration [72]. It allows large-scale analysis of RNA mixtures of potential therapeutic targets and cellular markers (http://cibersort.stanford.edu/; accessed on 15 May 2022). Subsequently, an unsupervised cluster analysis based on CIBERSORT calculations was performed to classify the immune infiltrate subtypes in the TCGA-STAD, GSE62254, GSE84437, and PRJEB25780 cohorts of patients to obtain immune cell infiltration (ICI) subtypes.

### 4.4. Building a Ferroptosis Score to Assess Individual Tumors

To more thoroughly examine the probable biological behavior among the ferroptosis molecular subtypes, overlapping differentially expressed genes (DEGs) between the ferroptosis subtypes were detected by the “limma” package (FDR < 0.001), and the prognosis was analyzed using a univariate Cox model (*p* < 0.05). Then, based on these DEGs, the patients were classified into genomic subtypes using an unsupervised clustering approach.

We used a principal component analysis (PCA) to quantify the ferroptosis molecular subtypes of individual tumors and to construct a scoring system that we termed the ferroptosis score [36,37]. We defined the ferroptosis score as follows: Ferroptosis score = ∑ (PC1i + PC2i), where i represents the expression of prognostic DEGs associated with the ferroptosis molecular subtypes. According to the threshold values determined by the “Survminer” R package, the patients were categorized into low and high ferroptosis groups.

### 4.5. Establishment and Validation of a Prognostic Model Based on Ferroptosis

The independent prognostic value of the ferroptosis score was determined with a univariate and multivariate Cox analysis. The diagnostic ROC curve and time-dependent ROC curve were employed to confirm the sensitivity and specificity of the ferroptosis score in predicting survival outcomes in GC. Next, a prediction model was constructed by integrating the ferroptosis score and other independent clinical risk factors according to the prognostic multivariate profile. A nomogram plot was used to visualize the relationship between the variables in the prediction model by following a certain scale in the same plane. A prognostic calibration plot was used for a fit analysis of the model to the actual situation and to determine the predictive efficacy. A risk factor plot was used to visualize how patients with gastric cancer were grouped by the median risk score of the multivariate Cox prediction model. The Prognostic DCA plot assessed the role of the prognostic model in terms of the clinical utility (patient benefit).

### 4.6. Immunotherapy Response Prediction and Chemotherapeutic Drug Sensitivity Prediction

In order to forecast the response to the immune checkpoint blockade (ICB), we assessed the tumor mutation burden (TMB). The “maftools” software package was used to visualize somatic mutation data extracted from the mutation annotation format (MAF) files [73]. For each patient, the TMB was estimated using the following formula: TMB = (total number of mutations)/(length of entire exon). In addition, the TIDE method was proposed by Jiang et al. in order to model the immune evasion mechanisms in cancer, including T-cell dysfunction and T-cell rejection [61]. In the present study, we used TIDE to assess the patient reactions to immunotherapy. Higher TIDE scores not only indicated a tumor with an immune evasion phenotype, but they also predicted a less favorable response to ICB in cancer patients.

The sensitivity of ferroptosis to chemotherapeutic agents was evaluated by the tumor drug sensitivity genomics (GDSC; https://www.cancerrxgene.org/; accessed on 20 May 2022) database [74]. The half maximum inhibitory concentration (IC_50_) was calculated by the “prophetic”.

### 4.7. Pan-Cancer Analysis: Ferroptosis Scores for 32 Cancers

We collected the gene expression information and relevant clinical data from the TCGA database for 32 tumor types. Over 11,000 tumor samples and normal tissues were scored for ferroptosis by a PCA analysis. The relationship between the ferroptosis scores and clinical characteristics (OS, DFS, DSS, PFS, Stage, TMB, and MSI) was further analyzed by a Spearman’s Rank Correlation.

### 4.8. Statistical Analysis

For comparisons between two groups, Wilcoxon rank sum tests were applied, while Kruskal–Wallis tests were employed for comparisons between multiple groups. The thresholds of each subgroup were calculated by “survminer”. We used Kaplan–Meier curves to compare the overall survival (OS) between subgroups and a log-rank test to determine. Using the maximum selected log-rank statistic, the ferroptosis scores were classified into high and low ferroptosis groups depending on the “surv-cutpoint”. The risk ratio (HR) of FRGs was calculated using a univariate Cox model.

## 5. Conclusions

In our study, we systematically investigated the molecular pattern of ferroptosis in GC and we proposed a ferroptosis score system, which is applicable to clinical practice to accurately assess the molecular subtypes of ferroptosis and corresponding TME characteristics within specific patients to further determine the immunophenotype and instruct more efficient clinical treatment. We also found that the ferroptosis score can be utilized as a separate prognostic factor to forecast patient survival. The clinical and pathological features of patients, such as stage, grade, histological subtypes, molecular subtypes, genetic variations, MSI status, and tumor mutation burden, could also be evaluated using this method. By the ferroptosis score, we also anticipated the efficacy of chemotherapy and the treatment response of patients to anti-PD-1/PD-L1 immunotherapy. This has provided some unique perspectives into immunotherapy for cancer, focusing on ferroptosis or ferroptosis molecular subtype-related genes to alter the ferroptosis scores and further ameliorate the unfavorable properties of TME. This research offers novel perspectives on how to enhance responses to immunotherapy, recognize different tumor immune characteristics, and advance individualized tumor immunotherapy in GC patients.

## Data Availability

Please contact the corresponding author to discuss availability of the data presented in this study.

## Supplementary Materials

The following supporting information can be downloaded at: https://www.mdpi.com/article/10.3390/ijms23179767/s1.

## Abbreviations

TME	tumor microenvironment
GC	gastric cancer
FRGs	ferroptosis-related genes
ROS	reactive oxygen species
CAFs	cancer-associated fibroblasts
TICs	tumor-associated immune cells
EMT	epithelial-mesenchymal transition
Pan-F-TBRS	pan-fibroblast TGF-β response signature
TMB	tumor mutational load
*GPX4*	glutathione peroxidase 4
CNVs	copy number variants
ICI	immune cell infiltration
CIBERSORT	cell-type identification by estimating relative subsets of RNA transcript
GSVA	gene set variation analysis
ssGSEA	Single Sample Gene Set Enrichment Analysis
GO	Gene ontology
KEGG	Kyoto Encyclopedia of Genes and Genomes
DEG	Differentially expressed gene
PCA	principal component analysis
ROC	Receiver operating characteristic
AUC	area under the curve
DCA	decision curve analysis
OS	overall survival
MSI	Microsatellite instability
EBV	EB virus infection
CIN	chromosomal instability
GS	genomic stability
MP	mesenchymal phenotype
EP	epithelial phenotype
ICB	immune checkpoint blockade
TIDE	Tumor Immune Dysfunction and Exclusion
IC_50_	the half maximum inhibitory concentration
DFS	disease-free survival
DSS	disease-specific survival
PFS	progression-free survival
MSS	Microsatellite stability
MAF	mutation annotation format
ESTIMATE	Estimation of Stromal and Immune Cells
HR	risk ratio
HPA	Human Protein Atlas

## References

[1] Sung H., Ferlay J., Siegel R.L., Laversanne M., Soerjomataram I., Jemal A., Bray F. (2021). Global Cancer Statistics 2020: GLOBOCAN Estimates of Incidence and Mortality Worldwide for 36 Cancers in 185 Countries. CA Cancer J. Clin..

[2] Smyth E.C., Nilsson M., Grabsch H.I., van Grieken N.C., Lordick F. (2020). Gastric cancer. Lancet.

[3] (2014). Comprehensive molecular characterization of gastric adenocarcinoma. Nature.

[4] Chao J., Fuchs C.S., Shitara K., Tabernero J., Muro K., Van Cutsem E., Bang Y.J., De Vita F., Landers G., Yen C.J. (2021). Assessment of Pembrolizumab Therapy for the Treatment of Microsatellite Instability-High Gastric or Gastroesophageal Junction Cancer Among Patients in the KEYNOTE-059, KEYNOTE-061, and KEYNOTE-062 Clinical Trials. JAMA Oncol..

[5] Janjigian Y.Y., Shitara K., Moehler M., Garrido M., Salman P., Shen L., Wyrwicz L., Yamaguchi K., Skoczylas T., Campos Bragagnoli A. (2021). First-line nivolumab plus chemotherapy versus chemotherapy alone for advanced gastric, gastro-oesophageal junction, and oesophageal adenocarcinoma (CheckMate 649): A randomised, open-label, phase 3 trial. Lancet.

[6] Kang Y.K., Boku N., Satoh T., Ryu M.H., Chao Y., Kato K., Chung H.C., Chen J.S., Muro K., Kang W.K. (2017). Nivolumab in patients with advanced gastric or gastro-oesophageal junction cancer refractory to, or intolerant of, at least two previous chemotherapy regimens (ONO-4538-12, ATTRACTION-2): A randomised, double-blind, placebo-controlled, phase 3 trial. Lancet.

[7] Shitara K., Özgüroğlu M., Bang Y.J., Di Bartolomeo M., Mandalà M., Ryu M.H., Fornaro L., Olesiński T., Caglevic C., Chung H.C. (2018). Pembrolizumab versus paclitaxel for previously treated, advanced gastric or gastro-oesophageal junction cancer (KEYNOTE-061): A randomised, open-label, controlled, phase 3 trial. Lancet.

[8] Binnewies M., Roberts E.W., Kersten K., Chan V., Fearon D.F., Merad M., Coussens L.M., Gabrilovich D.I., Ostrand-Rosenberg S., Hedrick C.C. (2018). Understanding the tumor immune microenvironment (TIME) for effective therapy. Nat. Med..

[9] Jiang Y., Zhang Q., Hu Y., Li T., Yu J., Zhao L., Ye G., Deng H., Mou T., Cai S. (2018). ImmunoScore Signature: A Prognostic and Predictive Tool in Gastric Cancer. Ann. Surg..

[10] Galluzzi L., Vitale I., Aaronson S.A., Abrams J.M., Adam D., Agostinis P., Alnemri E.S., Altucci L., Amelio I., Andrews D.W. (2018). Molecular mechanisms of cell death: Recommendations of the Nomenclature Committee on Cell Death 2018. Cell Death Differ..

[11] Chen X., Li J., Kang R., Klionsky D.J., Tang D. (2021). Ferroptosis: Machinery and regulation. Autophagy.

[12] Stockwell B.R., Jiang X., Gu W. (2020). Emerging Mechanisms and Disease Relevance of Ferroptosis. Trends Cell Biol..

[13] Friedmann Angeli J.P., Krysko D.V., Conrad M. (2019). Ferroptosis at the crossroads of cancer-acquired drug resistance and immune evasion. Nat. Rev. Cancer.

[14] Lei G., Zhuang L., Gan B. (2022). Targeting ferroptosis as a vulnerability in cancer. Nat. Rev. Cancer.

[15] Lang X., Green M.D., Wang W., Yu J., Choi J.E., Jiang L., Liao P., Zhou J., Zhang Q., Dow A. (2019). Radiotherapy and Immunotherapy Promote Tumoral Lipid Oxidation and Ferroptosis via Synergistic Repression of SLC7A11. Cancer Discov..

[16] Wang W., Green M., Choi J.E., Gijón M., Kennedy P.D., Johnson J.K., Liao P., Lang X., Kryczek I., Sell A. (2019). CD8(+) T cells regulate tumour ferroptosis during cancer immunotherapy. Nature.

[17] Zitvogel L., Kroemer G. (2019). Interferon-γ induces cancer cell ferroptosis. Cell Res..

[18] Bubnovskaya L., Osinsky D. (2020). Tumor microenvironment and metabolic factors: Contribution to gastric cancer. Exp. Oncol..

[19] Fridman W.H., Zitvogel L., Sautès-Fridman C., Kroemer G. (2017). The immune contexture in cancer prognosis and treatment. Nat. Rev. Clin. Oncol..

[20] Foroutan M., Cursons J., Hediyeh-Zadeh S., Thompson E.W., Davis M.J. (2017). A Transcriptional Program for Detecting TGFβ-Induced EMT in Cancer. Mol. Cancer Res..

[21] Xu Z., Feng J., Li Y., Guan D., Chen H., Zhai X., Zhang L., Li C., Li C. (2020). The vicious cycle between ferritinophagy and ROS production triggered EMT inhibition of gastric cancer cells was through p53/AKT/mTor pathway. Chem. Biol. Interact..

[22] Yuan X., Wu H., Han N., Xu H., Chu Q., Yu S., Chen Y., Wu K. (2014). Notch signaling and EMT in non-small cell lung cancer: Biological significance and therapeutic application. J. Hematol. Oncol..

[23] Damrauer J.S., Hoadley K.A., Chism D.D., Fan C., Tiganelli C.J., Wobker S.E., Yeh J.J., Milowsky M.I., Iyer G., Parker J.S. (2014). Intrinsic subtypes of high-grade bladder cancer reflect the hallmarks of breast cancer biology. Proc. Natl. Acad. Sci. USA.

[24] Hedegaard J., Lamy P., Nordentoft I., Algaba F., Høyer S., Ulhøi B.P., Vang S., Reinert T., Hermann G.G., Mogensen K. (2016). Comprehensive Transcriptional Analysis of Early-Stage Urothelial Carcinoma. Cancer Cell.

[25] Hugo W., Zaretsky J.M., Sun L., Song C., Moreno B.H., Hu-Lieskovan S., Berent-Maoz B., Pang J., Chmielowski B., Cherry G. (2016). Genomic and Transcriptomic Features of Response to Anti-PD-1 Therapy in Metastatic Melanoma. Cell.

[26] Sjödahl G., Lauss M., Lövgren K., Chebil G., Gudjonsson S., Veerla S., Patschan O., Aine M., Fernö M., Ringnér M. (2012). A molecular taxonomy for urothelial carcinoma. Clin. Cancer Res..

[27] Spranger S., Bao R., Gajewski T.F. (2015). Melanoma-intrinsic β-catenin signalling prevents anti-tumour immunity. Nature.

[28] Mariathasan S., Turley S.J., Nickles D., Castiglioni A., Yuen K., Wang Y., Kadel E.E., Koeppen H., Astarita J.L., Cubas R. (2018). TGFβ attenuates tumour response to PD-L1 blockade by contributing to exclusion of T cells. Nature.

[29] Zavros Y. (2017). Initiation and Maintenance of Gastric Cancer: A Focus on CD44 Variant Isoforms and Cancer Stem Cells. Cell. Mol. Gastroenterol. Hepatol..

[30] Jiang Y., Wang H., Wu J., Chen C., Yuan Q., Huang W., Li T., Xi S., Hu Y., Zhou Z. (2020). Noninvasive imaging evaluation of tumor immune microenvironment to predict outcomes in gastric cancer. Ann. Oncol..

[31] Gu R., Xia Y., Li P., Zou D., Lu K., Ren L., Zhang H., Sun Z. (2022). Ferroptosis and its Role in Gastric Cancer. Front. Cell Dev. Biol..

[32] Li W., Feng G., Gauthier J.M., Lokshina I., Higashikubo R., Evans S., Liu X., Hassan A., Tanaka S., Cicka M. (2019). Ferroptotic cell death and TLR4/Trif signaling initiate neutrophil recruitment after heart transplantation. J. Clin. Invest..

[33] Viswanathan V.S., Ryan M.J., Dhruv H.D., Gill S., Eichhoff O.M., Seashore-Ludlow B., Kaffenberger S.D., Eaton J.K., Shimada K., Aguirre A.J. (2017). Dependency of a therapy-resistant state of cancer cells on a lipid peroxidase pathway. Nature.

[34] Chen D.S., Mellman I. (2017). Elements of cancer immunity and the cancer-immune set point. Nature.

[35] Pontén F., Schwenk J.M., Asplund A., Edqvist P.H. (2011). The Human Protein Atlas as a proteomic resource for biomarker discovery. J. Intern. Med..

[36] Sotiriou C., Wirapati P., Loi S., Harris A., Fox S., Smeds J., Nordgren H., Farmer P., Praz V., Haibe-Kains B. (2006). Gene expression profiling in breast cancer: Understanding the molecular basis of histologic grade to improve prognosis. J. Natl. Cancer Inst..

[37] Zhang X., Shi M., Chen T., Zhang B. (2020). Characterization of the Immune Cell Infiltration Landscape in Head and Neck Squamous Cell Carcinoma to Aid Immunotherapy. Mol. Nucleic Acids.

[38] Cristescu R., Lee J., Nebozhyn M., Kim K.M., Ting J.C., Wong S.S., Liu J., Yue Y.G., Wang J., Yu K. (2015). Molecular analysis of gastric cancer identifies subtypes associated with distinct clinical outcomes. Nat. Med..

[39] Asaoka Y., Ijichi H., Koike K. (2015). PD-1 Blockade in Tumors with Mismatch-Repair Deficiency. N. Engl. J. Med..

[40] Samstein R.M., Lee C.H., Shoushtari A.N., Hellmann M.D., Shen R., Janjigian Y.Y., Barron D.A., Zehir A., Jordan E.J., Omuro A. (2019). Tumor mutational load predicts survival after immunotherapy across multiple cancer types. Nat. Genet..

[41] Oh S.C., Sohn B.H., Cheong J.H., Kim S.B., Lee J.E., Park K.C., Lee S.H., Park J.L., Park Y.Y., Lee H.S. (2018). Clinical and genomic landscape of gastric cancer with a mesenchymal phenotype. Nat. Commun..

[42] Barbie D.A., Tamayo P., Boehm J.S., Kim S.Y., Moody S.E., Dunn I.F., Schinzel A.C., Sandy P., Meylan E., Scholl C. (2009). Systematic RNA interference reveals that oncogenic KRAS-driven cancers require TBK1. Nature.

[43] Zeng D., Li M., Zhou R., Zhang J., Sun H., Shi M., Bin J., Liao Y., Rao J., Liao W. (2019). Tumor Microenvironment Characterization in Gastric Cancer Identifies Prognostic and Immunotherapeutically Relevant Gene Signatures. Cancer Immunol. Res..

[44] Louandre C., Ezzoukhry Z., Godin C., Barbare J.C., Mazière J.C., Chauffert B., Galmiche A. (2013). Iron-dependent cell death of hepatocellular carcinoma cells exposed to sorafenib. Int. J. Cancer.

[45] Louandre C., Marcq I., Bouhlal H., Lachaier E., Godin C., Saidak Z., François C., Chatelain D., Debuysscher V., Barbare J.C. (2015). The retinoblastoma (Rb) protein regulates ferroptosis induced by sorafenib in human hepatocellular carcinoma cells. Cancer Lett..

[46] Guo J., Xu B., Han Q., Zhou H., Xia Y., Gong C., Dai X., Li Z., Wu G. (2018). Ferroptosis: A Novel Anti-tumor Action for Cisplatin. Cancer Res. Treat..

[47] Xu H., Ye D., Ren M., Zhang H., Bi F. (2021). Ferroptosis in the tumor microenvironment: Perspectives for immunotherapy. Trends Mol. Med..

[48] Jiang Y., Xie J., Huang W., Chen H., Xi S., Han Z., Huang L., Lin T., Zhao L.Y., Hu Y.F. (2019). Tumor Immune Microenvironment and Chemosensitivity Signature for Predicting Response to Chemotherapy in Gastric Cancer. Cancer Immunol. Res..

[49] Nishino M., Ramaiya N.H., Hatabu H., Hodi F.S. (2017). Monitoring immune-checkpoint blockade: Response evaluation and biomarker development. Nat. Rev. Clin. Oncol..

[50] Sui S., Xu S., Pang D. (2022). Emerging role of ferroptosis in breast cancer: New dawn for overcoming tumor progression. Pharm. Ther..

[51] Pan F., Lin X., Hao L., Wang T., Song H., Wang R. (2022). The Critical Role of Ferroptosis in Hepatocellular Carcinoma. Front. Cell Dev. Biol..

[52] Zhang W., Jiang B., Liu Y., Xu L., Wan M. (2022). Bufotalin induces ferroptosis in non-small cell lung cancer cells by facilitating the ubiquitination and degradation of GPX4. Free Radic. Biol. Med..

[53] Liu T., Zhu C., Chen X., Guan G., Zou C., Shen S., Wu J., Wang Y., Lin Z., Chen L. (2022). Ferroptosis, as the most enriched programmed cell death process in glioma, induces immunosuppression and immunotherapy resistance. Neuro Oncol..

[54] Chen X., Kang R., Kroemer G., Tang D. (2021). Broadening horizons: The role of ferroptosis in cancer. Nat. Rev. Clin. Oncol..

[55] Hegde P.S., Karanikas V., Evers S. (2016). The Where, the When, and the How of Immune Monitoring for Cancer Immunotherapies in the Era of Checkpoint Inhibition. Clin. Cancer Res..

[56] Tauriello D.V.F., Palomo-Ponce S., Stork D., Berenguer-Llergo A., Badia-Ramentol J., Iglesias M., Sevillano M., Ibiza S., Cañellas A., Hernando-Momblona X. (2018). TGFβ drives immune evasion in genetically reconstituted colon cancer metastasis. Nature.

[57] Charoentong P., Finotello F., Angelova M., Mayer C., Efremova M., Rieder D., Hackl H., Trajanoski Z. (2017). Pan-cancer Immunogenomic Analyses Reveal Genotype-Immunophenotype Relationships and Predictors of Response to Checkpoint Blockade. Cell Rep..

[58] Cristescu R., Mogg R., Ayers M., Albright A., Murphy E., Yearley J., Sher X., Liu X.Q., Lu H., Nebozhyn M. (2018). Pan-tumor genomic biomarkers for PD-1 checkpoint blockade-based immunotherapy. Science.

[59] Kandoth C., McLellan M.D., Vandin F., Ye K., Niu B., Lu C., Xie M., Zhang Q., McMichael J.F., Wyczalkowski M.A. (2013). Mutational landscape and significance across 12 major cancer types. Nature.

[60] Jiang P., Gu S., Pan D., Fu J., Sahu A., Hu X., Li Z., Traugh N., Bu X., Li B. (2018). Signatures of T cell dysfunction and exclusion predict cancer immunotherapy response. Nat. Med..

[61] Kim S.T., Cristescu R., Bass A.J., Kim K.M., Odegaard J.I., Kim K., Liu X.Q., Sher X., Jung H., Lee M. (2018). Comprehensive molecular characterization of clinical responses to PD-1 inhibition in metastatic gastric cancer. Nat. Med..

[62] Zhou N., Bao J. (2020). FerrDb: A manually curated resource for regulators and markers of ferroptosis and ferroptosis-disease associations. Database (Oxf. ).

[63] Colaprico A., Silva T.C., Olsen C., Garofano L., Cava C., Garolini D., Sabedot T.S., Malta T.M., Pagnotta S.M., Castiglioni I. (2016). TCGAbiolinks: An R/Bioconductor package for integrative analysis of TCGA data. Nucleic Acids Res..

[64] Ritchie M.E., Phipson B., Wu D., Hu Y., Law C.W., Shi W., Smyth G.K. (2015). limma powers differential expression analyses for RNA-sequencing and microarray studies. Nucleic Acids Res..

[65] Wilkerson M.D., Hayes D.N. (2010). ConsensusClusterPlus: A class discovery tool with confidence assessments and item tracking. Bioinformatics.

[66] Monti S., Tamayo P., Mesirov J.P., Golub T.R. (2003). Consensus Clustering: A Resampling-Based Method for Class Discovery and Visualization of Gene Expression Microarray Data. Mach. Learn..

[67] Yu G., Wang L.G., Han Y., He Q.Y. (2012). clusterProfiler: An R package for comparing biological themes among gene clusters. Omics.

[68] Hänzelmann S., Castelo R., Guinney J. (2013). GSVA: Gene set variation analysis for microarray and RNA-seq data. BMC Bioinform..

[69] Subramanian A., Tamayo P., Mootha V.K., Mukherjee S., Ebert B.L., Gillette M.A., Paulovich A., Pomeroy S.L., Golub T.R., Lander E.S. (2005). Gene set enrichment analysis: A knowledge-based approach for interpreting genome-wide expression profiles. Proc. Natl. Acad. Sci. USA.

[70] McFaline-Figueroa J.L., Hill A.J., Qiu X., Jackson D., Shendure J., Trapnell C. (2019). A pooled single-cell genetic screen identifies regulatory checkpoints in the continuum of the epithelial-to-mesenchymal transition. Nat. Genet..

[71] Yoshihara K., Shahmoradgoli M., Martínez E., Vegesna R., Kim H., Torres-Garcia W., Treviño V., Shen H., Laird P.W., Levine D.A. (2013). Inferring tumour purity and stromal and immune cell admixture from expression data. Nat. Commun..

[72] Newman A.M., Liu C.L., Green M.R., Gentles A.J., Feng W., Xu Y., Hoang C.D., Diehn M., Alizadeh A.A. (2015). Robust enumeration of cell subsets from tissue expression profiles. Nat. Methods.

[73] Mayakonda A., Lin D.C., Assenov Y., Plass C., Koeffler H.P. (2018). Maftools: Efficient and comprehensive analysis of somatic variants in cancer. Genome Res..

[74] Yang W., Soares J., Greninger P., Edelman E.J., Lightfoot H., Forbes S., Bindal N., Beare D., Smith J.A., Thompson I.R. (2013). Genomics of Drug Sensitivity in Cancer (GDSC): A resource for therapeutic biomarker discovery in cancer cells. Nucleic Acids Res..

