# Supplementary figures and images for: The Ferroptosis Molecular Subtype Reveals Characteristics of the Tumor Microenvironment, Immunotherapeutic Response, and Prognosis in Gastric Cancer
Source: Int J Mol Sci. 2022 Aug 29;23(17):9767. doi: 10.3390/ijms23179767 (PMC9456108; doi:10.3390/ijms23179767)

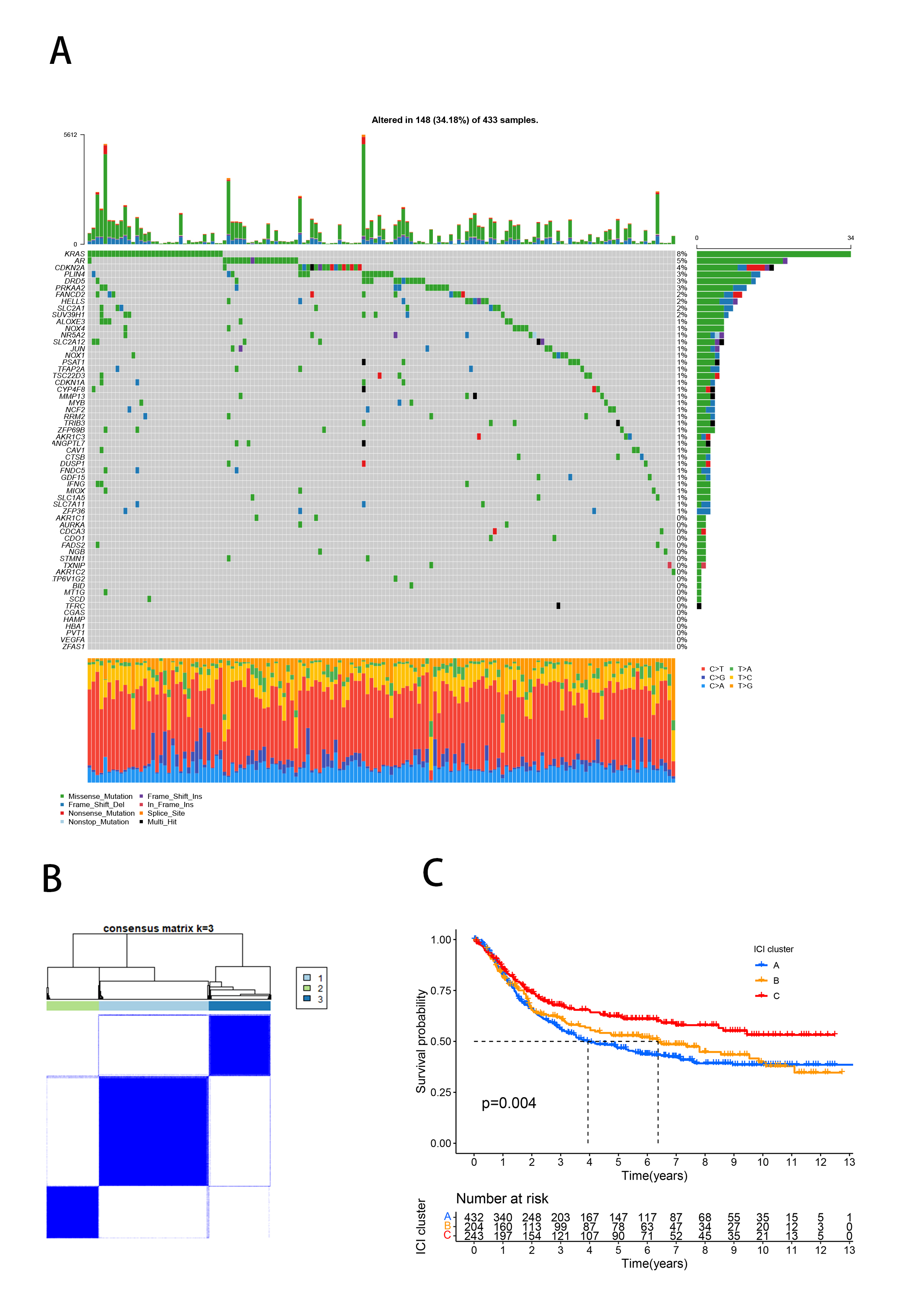

Supplement: Supplementary file 1 [file ijms-23-09767-s001.zip › S1.tif]

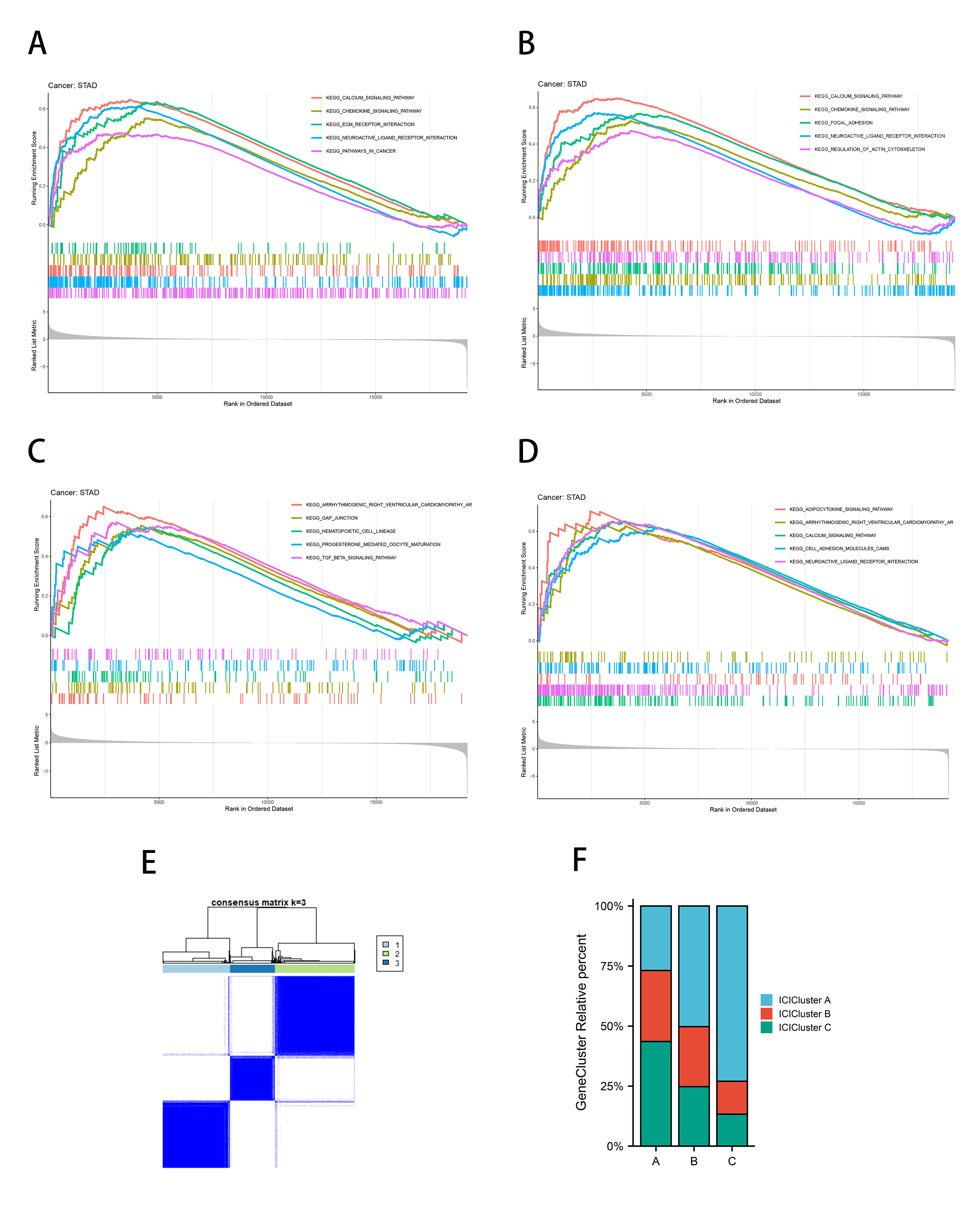

Supplement: Supplementary file 1 [file ijms-23-09767-s001.zip › S2.tif]

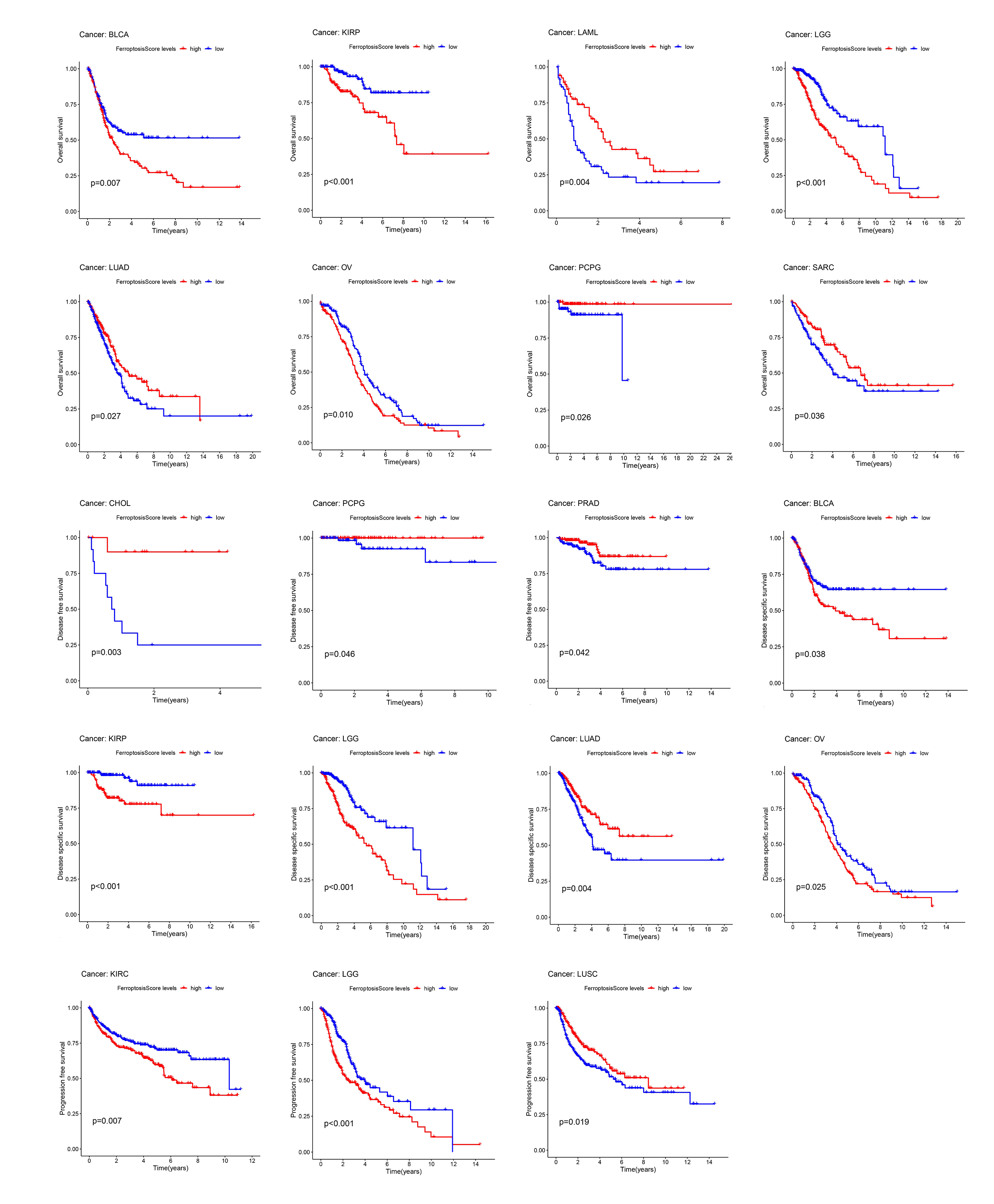

Supplement: Supplementary file 1 [file ijms-23-09767-s001.zip › S3.tif]

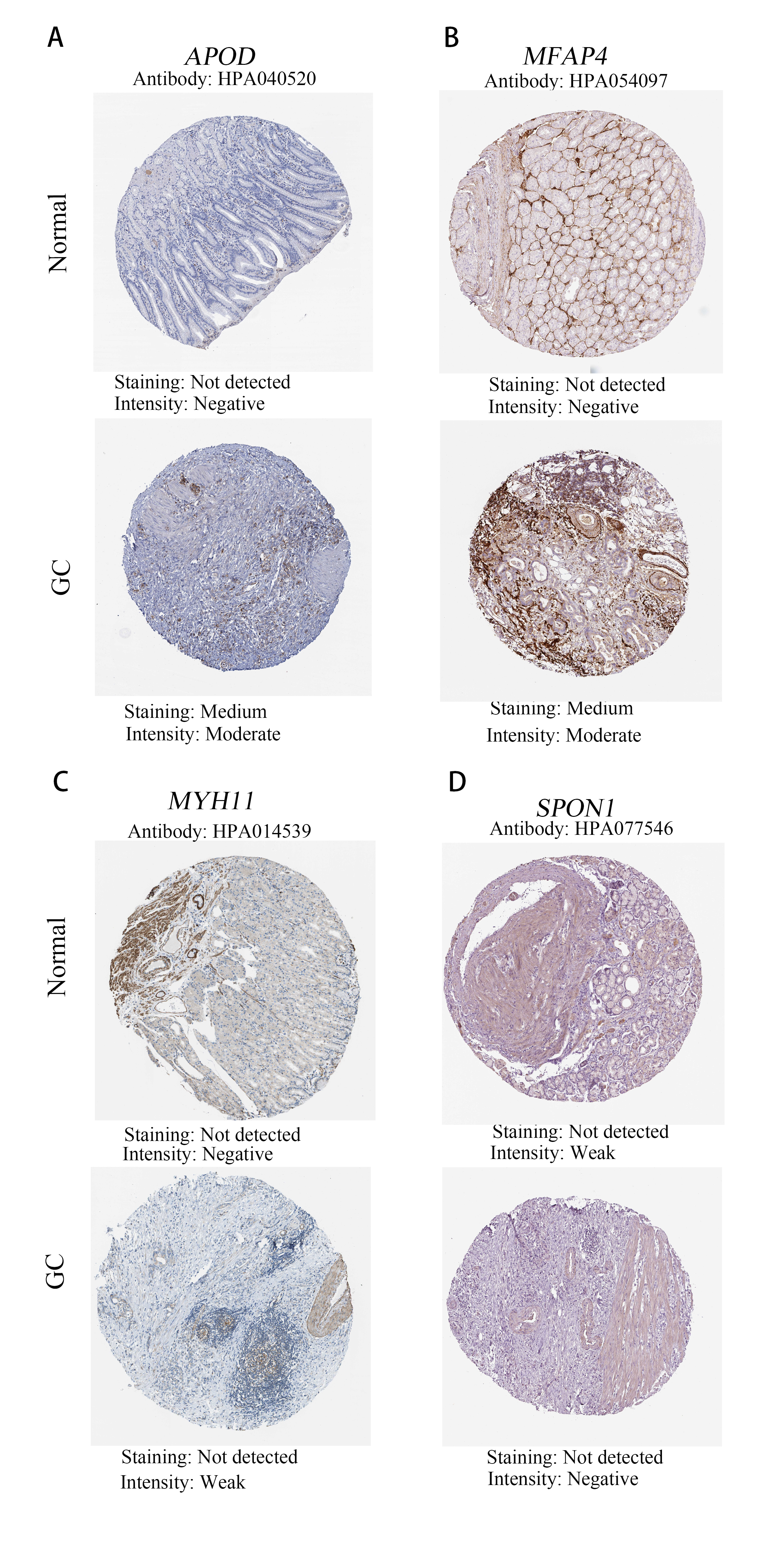

Supplement: Supplementary file 1 [file ijms-23-09767-s001.zip › S4.tif]
